# Evaluation of a Novel e-Learning Program for Physiotherapists to Manage Knee Osteoarthritis via Telehealth: Qualitative Study Nested in the PEAK (Physiotherapy Exercise and Physical Activity for Knee Osteoarthritis) Randomized Controlled Trial

**DOI:** 10.2196/25872

**Published:** 2021-04-30

**Authors:** Sarah E Jones, Penny K Campbell, Alexander J Kimp, Kim Bennell, Nadine E Foster, Trevor Russell, Rana S Hinman

**Affiliations:** 1 Department of Physiotherapy, School of Health Sciences Centre for Health, Exercise and Sports Medicine The University of Melbourne Melbourne Australia; 2 Primary Care Centre Versus Arthritis School of Medicine Keele University Keele United Kingdom; 3 STARS Education and Research Alliance School of Health and Behavioural Sciences The University of Queensland Brisbane Australia; 4 RECOVER Injury Research Centre The University of Queensland Brisbane Australia

**Keywords:** osteoarthritis, knee, physiotherapy, exercise, e-learning, qualitative, telehealth, pain, education

## Abstract

**Background:**

The delivery of physiotherapy via telehealth could provide more equitable access to services for patients. Videoconference-based telehealth has been shown to be an effective and acceptable mode of service delivery for exercise-based interventions for chronic knee pain; however, specific training in telehealth is required for physiotherapists to effectively and consistently deliver care using telehealth. The development and evaluation of training programs to upskill health care professionals in the management of osteoarthritis (OA) has also been identified as an important priority to improve OA care delivery.

**Objective:**

This study aims to explore physiotherapists’ experiences with and perceptions of an e-learning program about best practice knee OA management (focused on a structured program of education, exercise, and physical activity) that includes telehealth delivery via videoconferencing.

**Methods:**

We conducted a qualitative study using individual semistructured telephone interviews, nested within the *Physiotherapy Exercise and Physical Activity for Knee Osteoarthritis* randomized controlled trial, referred to as the *PEAK* trial. A total of 15 Australian physiotherapists from metropolitan and regional private practices were interviewed following the completion of an e-learning program. The *PEAK* trial e-learning program involved self-directed learning modules, a mock video consultation with a researcher (simulated patient), and 4 audited practice video consultations with pilot patients with chronic knee pain. Interviews were audio recorded and transcribed verbatim. Data were thematically analyzed.

**Results:**

A total of five themes (with associated subthemes) were identified: the experience of self-directed e-learning (physiotherapists were more familiar with in-person learning; however, they valued the comprehensive, self-paced web-based modules. Unwieldy technological features could be frustrating); practice makes perfect (physiotherapists benefited from the mock consultation with the researcher and practice sessions with pilot patients alongside individualized performance feedback, resulting in confidence and preparedness to implement new skills); the telehealth journey (although inexperienced with telehealth before training, physiotherapists were confident and able to deliver remote care following training; however, they still experienced some technological challenges); the *whole package* (the combination of self-directed learning modules, mock consultation, and practice consultations with pilot patients was felt to be an effective learning approach, and patient information booklets supported the training package); and impact on broader clinical practice (training consolidated and refined existing OA management skills and enabled a switch to telehealth when the COVID-19 pandemic affected in-person clinical care).

**Conclusions:**

Findings provide evidence for the perceived effectiveness and acceptability of an e-learning program to train physiotherapists (in the context of a clinical trial) on best practice knee OA management, including telehealth delivery via videoconferencing. The implementation of e-learning programs to upskill physiotherapists in telehealth appears to be warranted, given the increasing adoption of telehealth service models for the delivery of clinical care.

## Introduction

### Background

Physiotherapy care is traditionally delivered via in-person consultations. However, for many people, access to physiotherapy is limited by geographical isolation, or limited local services, or both [1]. To this end, experts in Australia [2] and the United Kingdom National Health Service [3] have identified the need to make full use of digital technologies to facilitate patient convenience and access to care. Such services have become even more critical during the COVID-19 pandemic. Social distancing requirements and *lockdown* restrictions have affected the delivery of in-person health care worldwide, including for noncommunicable diseases, where rehabilitation services have been among the hardest hit [4]. The COVID-19 pandemic has accelerated the drive toward telehealth service delivery as a safe and viable model for physiotherapy services [5,6].

Telehealth is defined by the World Health Organization as the “delivery of health care services, where patients and providers are separated by distance. Telehealth uses information communication technology for the exchange of information for the diagnosis and treatment of diseases and injuries, research and evaluation, and for the continuing education of health professionals” [7]. Data suggest that only a minority of physiotherapists were providing telehealth services prepandemic [8,9], highlighting the lack of telehealth experience within the profession. Delivering physiotherapy via telehealth requires new technical skills and new clinical skills to adapt clinical practice to treating a patient located remotely from the clinician [10,11]. Although generally computer literate, clinicians delivering rehabilitation services for chronic pain typically have limited confidence and knowledge in the use of telehealth [12]. Furthermore, clinician acceptance and confidence in telehealth increases with both training and repeated exposure to telehealth practice [10,12,13]. Thus, specific training in telehealth is required for physiotherapists to effectively and consistently deliver care via this medium [14].

Advancing technologies allow education and training for medical and allied health students and professionals to overcome learning barriers such as distance and the limited availability of specialist training staff while also providing a standardized experience for learners [15]. e-Learning broadly relates to the delivery of educational material through information and communication technology (ICT) [16,17], using the internet to wholly or partially replace the need for a human instructor [18,19]. e-Learning can be synchronous (mediated in real time, eg, videoconference), asynchronous (self-directed, self-paced learning), or a combination of both. A systematic review of studies in practicing or trainee doctors and other health professionals compared e-learning interventions with noninternet learning or no learning interventions. Findings revealed that e-learning was associated with large positive effects on education outcomes (knowledge and skills) compared with no learning, whereas only small, inconsistent effects were seen between e-learning and noninternet learning [18], suggesting that e-learning may be similarly effective to more traditional teaching methods.

Osteoarthritis (OA) is a common and often debilitating chronic joint disease and is one of the leading causes of pain and disability in Australia [20] and worldwide [21]. Symptoms can become increasingly debilitating over time and can greatly affect quality of life, contributing to feelings of dependence and loss of autonomy in older people [22]. As a result of an aging population, combined with increasing obesity rates, the disease burden associated with knee OA is forecast to increase substantially over the coming decade [23]. There is no cure for OA. However, improvements in pain, physical function, and quality of life have been demonstrated with exercise-based interventions [24,25]. Thus, clinical guidelines consistently emphasize education, exercise, weight loss (if required), and support for self-management to alleviate knee OA symptoms before using surgical or pharmacological interventions [26-30].

Given the central role of exercise in disease management, physiotherapists in primary care settings play an important role in providing care to people with knee OA. However, physiotherapists often feel underprepared to manage OA, lacking knowledge about evidence-based practice and confidence in implementing recommendations into routine care [31-34]. The development and evaluation of training programs to upskill health care professionals in OA management has thus been identified as an important priority for improving OA care [35-37]. Telephone- and videoconference-based telehealth interventions have been shown to be effective and acceptable [14,38-40] modes of service delivery for exercise-based interventions aimed at relieving chronic knee pain and improving physical dysfunction and are as effective as in-person care for adults with musculoskeletal pain [41]. Australia’s National Osteoarthritis Strategy [20] has also called for the increased implementation of remotely delivered evidence-based OA services.

### Objectives

In this study, we explore the experiences of physiotherapists with and their perceptions of an e-learning program aimed at educating physiotherapists about best practice knee OA management, including the implementation of a protocolized management program focused on education, exercise, and physical activity, and how to deliver such care remotely using a videoconferencing platform.

## Methods

### Design

A qualitative study nested within an ongoing randomized controlled trial (RCT; Australian New Zealand Clinical Trials Registry: ACTRN12619001240134) [42] was conducted. The Physiotherapy Exercise and Physical Activity for Knee OA RCT (known and referred to as the *PEAK* trial) is a noninferiority trial comparing physiotherapist-delivered in-person consultations with physiotherapist-delivered video consultations for people with knee OA. For the RCT, 15 physiotherapists were provided with a structured program of e-learning in best practice knee OA management, including the implementation of a protocolized management program focused on education, exercise, and physical activity as well as how to deliver such care remotely using a videoconferencing platform. In this study, a qualitative approach was chosen to explore the physiotherapist’s experience of e-learning as well as its impact and perceived effectiveness. The research design was centered around a constructivist paradigm, which asserts that people generate their own understanding and knowledge subjectively through experience and reflection [43]. The Standards for Reporting Qualitative Research checklist guided the reporting [44].

### Participants

All 15 physiotherapists recruited to deliver trial interventions for the PEAK RCT participated in this qualitative study. Eligibility criteria for physiotherapist participation in the RCT included current registration to practice as a physiotherapist, private practice located in metropolitan or regional Victoria or Queensland (Australia), access to a computer with internet connection, suitable workspace for confidential video consultations, and some previous experience with videoconferencing software, for example, Skype, Zoom, or Facetime (not necessarily for delivering clinical care). Physiotherapists who met the eligibility criteria were considered for the study, and participating physiotherapists were selected based on their availability and location to ensure geographical spread across metropolitan and regional areas of Victoria and Queensland. All participants provided written informed consent, and the qualitative study was approved by the Institutional Human Research Ethics Committee, separate from the RCT. 

### Training Program

**Figure 1:**
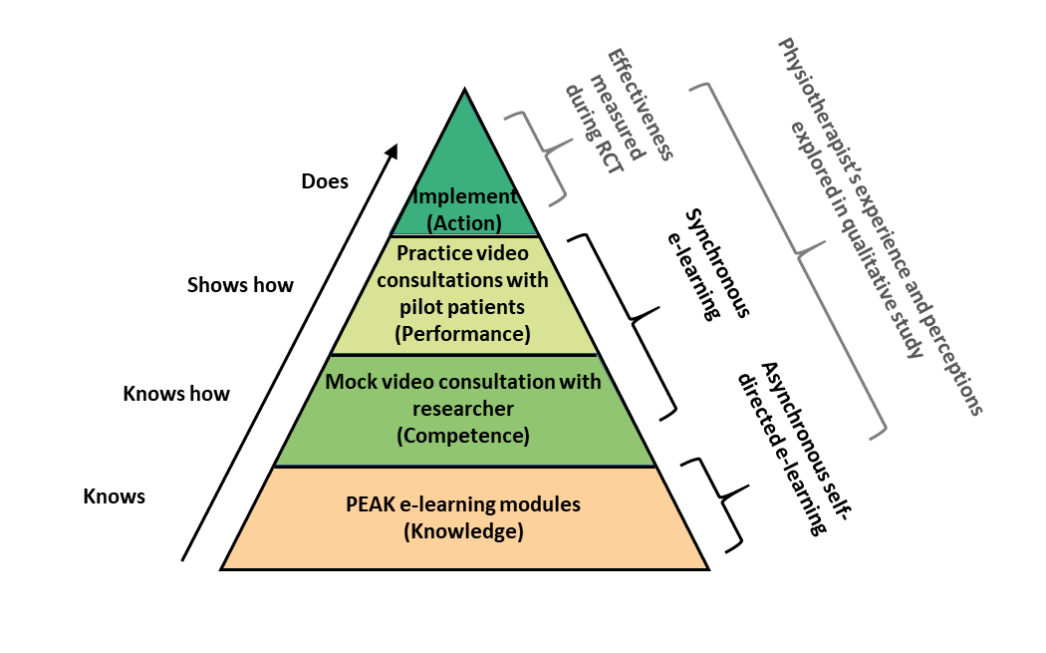
Schematic representation of the PEAK (Physiotherapy Exercise and Physical Activity for Knee Osteoarthritis) training components mapped to the Miller Pyramid learning model. PEAK: Physiotherapy Exercise and Physical Activity for Knee Osteoarthritis. RCT: randomized controlled trial.

The PEAK trial training program was developed by the research team, using an e-learning approach featuring both asynchronous and synchronous learning, developed in line with the Miller Pyramid (Figure 1), a learning model designed for use in health education [45]. The model proposes that the foundation of the pyramid is knowledge (*knows*), with learners understanding the relevant facts and theories. The next level is competence (*knows how*), with learners developing skills necessary to apply knowledge, demonstrated via a mock video consultation with a researcher (simulated patient). Next is performance (*shows how*), with learners demonstrating, via practice video consultations with pilot patients, that they can apply knowledge and skills in a realistic clinical context. The final stage represents action (*does*)—the stage at which the learner has integrated their knowledge, skills, and attitudes and is prepared to integrate the competency into their clinical practice. Results from the linked RCT will provide data on the effectiveness of the implementation.

Physiotherapists first completed approximately 5 hours of self-directed e-learning modules delivered at the University of Melbourne Learning Management System (LMS; Canvas LMS by Instructure, 2019) covering OA management best practices (including a structured physiotherapy treatment protocol), telehealth (the delivery of care via Zoom videoconferencing), and trial procedures. Each module included a quiz to help reinforce user’s knowledge and learning. e-Learning was completed at the user’s own self-selected pace (ideally within 4 weeks) and delivered knowledge as the foundation of the physiotherapist’s learning. The PEAK training program e-learning modules have since been released for use by clinicians more widely [46].

The e-learning then moved to *practical* synchronous components, whereby physiotherapists participated in a mock initial consultation via videoconferencing with a physiotherapist researcher (AJK; simulated patient), who provided immediate verbal feedback on consultation quality and performance and evaluated the physiotherapist’s competency according to a standardized competency checklist (Multimedia Appendix 1). The next stage of *practical* synchronous e-learning required physiotherapists to complete 4 practice video consultations, with 2 pilot patients with chronic (>3 months) knee pain (recruited by research staff) to practice their video consultation skills and apply the education, exercise, and physical activity management program taught in the e-learning modules in a realistic scenario. The research staff conducted spot-checks of consultation recordings to assess the fidelity of the practice consultations to the protocolized treatment plan. Written feedback on common mistakes was provided to participants, and personalized feedback was provided verbally to individual physiotherapists after all pilot patient consultations had been completed. Physiotherapists were financially compensated for the time away from private practice that was dedicated to all elements of participating in the training program and the time taken to complete the interviews.

### Semistructured Interviews

Semistructured interviews were conducted after physiotherapists completed all components of the training program and commenced treating participants in the PEAK RCT. The interview schedule (Table 1) was developed based on a constructivist schema whereby physiotherapist expectations, training experience, and perceptions of effectiveness of the e-learning program in changing confidence, imparting knowledge, and addressing learning needs were explored. Questions were loosely aligned with the theoretical framework of acceptability [47], focusing on the framework’s five most relevant constructs to the e-learning program (affective attitude, burden, perceived effectiveness, intervention coherence, and self-efficacy). All interviews were conducted over the telephone by SEJ (a researcher who is not a physiotherapist but is trained in qualitative research) who was not involved in the RCT or in developing the training program and was otherwise unknown to the participants. Interviews lasted approximately 30 minutes and were audio recorded and then transcribed verbatim by a third party. Audio recordings and transcripts were deidentified, with transcripts assigned gender-matched pseudonyms to ensure participant confidentiality. All data were stored in digital format on a password-protected university server.

**Table 1:** Semistructured interview schedule (loosely aligned with the theoretical framework of acceptability).

Topic and construct			Questions (prompts)
**Expectations**			
	**Affective attitude; self-efficacy**		
		1. Can you tell me how confident you were in your ability to deliver an OA^a^ management program before the PEAK^b^ trial training? Previous OA management training? *Usual* management of knee OA before training?	
		2. Can you tell me about how confident you were in your ability to deliver physiotherapy to OA patients via videoconferencing, before you started the PEAK training? Training or professional development or experience in telehealth?	
		3. What were your overall impressions and experience of the training program? Expectations? Comparison with previous professional development?	
**Training experience**			
	**Burden**		
		4. What did you like and dislike about the training program? Consider both the web-based modules and the *practical* aspects of training (ie, the *competency* video consultation and 4 practice patient video consultations)? Time taken for web-based modules? Volume of material?	
	**Intervention coherence**		
		5. To what extent did you feel your learning needs were met (or not) through the PEAK training program? Was there anything else you felt need to be covered or a required a greater focus in the training? Was there anything included that you felt was unnecessary?	
		6. What were your thoughts on the content and presentation of the content in the PEAK web-based modules? How did you feel about the volume of text presented? How would you prefer to see the content delivered? If you were to change the content or its presentation, what would you suggest?	
		7. What were your impressions about the usability of the web-based training platform? Like/dislikes/changes?	
		8. How did you find the *competency video consultation* with the research staff member? Was it useful?	
		9. Can you tell me a little bit about what you thought about the practice video consultation sessions with the pilot patients with knee pain? Were these consultations useful?	
	**Perceived effectiveness**		
		10. Can you tell me overall how useful you found the PEAK trial training program? Intent to implement changes to usual clinical practice?	
		11. How confident do you feel in delivering OA management to patients now, after completing the PEAK trial training? What elements do you feel most/least confident with?	
		12. How confident do you feel in delivering physiotherapy to your OA patients over videoconference, now that you have completed the PEAK training? What elements do you feel most/least confident with?	
**Concluding remarks**			
		13. Given the restrictions on clinical practice imposed by the current COVID-19 pandemic, has the PEAK training program enabled you to make any changes to your own current clinical practice?	
		14. Thank you very much for all your time today and all your time and efforts with this study. Is there anything else you would like to add about your experiences with the PEAK training program?	

^a^OA: osteoarthritis.

^b^PEAK: Physiotherapy Exercise and Physical Activity for Knee Osteoarthritis.

### Data Analysis

Data analysis was conducted using a thematic approach [48]. Shortly after transcription, interview transcripts were read by SEJ to familiarize with the data. Transcripts were then reread and coded, with text indexed into topics, each identified with a short descriptor. To demonstrate the credibility and confirmability of the emergent topics and patterns, coding was also performed by a second researcher PKC (not a physiotherapist but assisted with developing the training program and recruited physiotherapists into the RCT, and trained in qualitative research). Topics identified by both SEJ and PKC were reviewed, and in collaboration, closely related topics were collated to generate emergent themes within the data. All transcripts were also read by RSH (a physiotherapist who led the development of the training program and an experienced qualitative researcher) who confirmed the relevance of emergent themes across transcripts. Emergent themes were then further refined, ensuring clear and encompassing definitions were generated when naming final themes and subthemes. Themes and subthemes were presented with exemplary quotes from interviews to demonstrate the transferability of the results [49].

## Results

### Physiotherapist Characteristics

All 15 physiotherapists recruited to deliver care as part of the PEAK RCT were invited to participate in the qualitative interviews. The sample comprised physiotherapists who worked in private practices across 2 Australian states. Physiotherapist characteristics are summarized in Table 2, including the number of PEAK RCT participant consultations conducted by each physiotherapist at the time of the interview. Participating physiotherapists were more often male (11/15, 73%). There was an even divide between those who worked in major cities (8/15, 53%) and those who worked in regional areas (7/15, 47%). Of the 14 physiotherapists, 8 of them (60%) had no previous experience with telehealth. The mean (SD) of years of clinical experience was 11 years (SD 4), and the mean numbers of PEAK RCT participants who physiotherapists had already treated at the time of the interview were 2 (SD 1) via videoconferencing and 2 (SD 2) in-person.

**Table 2:** Physiotherapists’ characteristics (n=15).

Pseudonym	Sex	Geographical location^a^	Clinical experience (years)	Previous experience with telehealth	PEAK^b^ RCT^c^ videoconferencing participants at the time of the interview, n	PEAK RCT in-person participants at the time of the interview, n
Daniel	Male	Outer regional, Victoria	19	Yes	1	0
Edward	Male	Major city, Queensland	10	No	1	0
Gregory	Male	Major city, Victoria	4	No	0	0
Jason	Male	Inner regional, Queensland	12	Yes	5	2
Robert	Male	Outer regional, Victoria	12	No	2	2
Steven	Male	Inner regional, Victoria	9	No	2	3
Nicole	Female	Inner regional, Victoria	15	No	3	5
Anthony	Male	Major city, Queensland	6	Yes	2	3
Mark	Male	Major city, Queensland	9	Yes	2	3
Douglas	Male	Major city, Victoria	18	No	4	3
Caroline	Female	Major city, Victoria	7	No	1	4
Leslie	Female	Major city, Queensland	9	No	0	1
Brian	Male	Inner regional, Queensland	14	Yes	3	0
William	Male	Major city, Victoria	10	No	1	1
Vicki	Female	Outer regional, Queensland	5	Yes	1	4

^a^Level of remoteness, based on residential postcode, in accordance with the Australian Statistical Geographical Classification-Remoteness Area.

^b^PEAK: Physiotherapy Exercise and Physical Activity for Knee Osteoarthritis.

^c^RCT: randomized controlled trial.

### Emergent Themes

A total of five themes, with associated subthemes, emerged and are described in Multimedia Appendix 2.

#### Theme 1: The Experience of Self-Directed e-Learning

Physiotherapists found the self-directed e-learning modules to be of high quality, comprehensive, and user-friendly and that they would “benefit the least experienced physiotherapist and the most experienced physiotherapist”. Although physiotherapists were more familiar with “hands-on” in-person professional development training for furthering their knowledge and clinical skills, they highly valued the web-based structure. In particular, the self-paced nature of the program was highly regarded and enabled the physiotherapists to fit the training into their busy daily lives and complete it as time allowed. However, the physiotherapists spoke of feeling annoyed and frustrated by some unwieldy features of the delivery platform (including log-in and navigation). Although most physiotherapists spoke highly of the web-based modules, 3 physiotherapists with divergent views spoke of the e-learning modules being “slow going”, “tedious”, or “fatiguing”. The telehealth learning module content, in particular, was felt to be “dry” and “overtly obvious”.

#### Theme 2: Practice Makes Perfect

Physiotherapists frequently referenced the benefit gained from individualized performance feedback from the research team during or after the practical components of the training. They spoke of “learning from applying” and that feedback was helpful ahead of implementing their new skills in a true clinical scenario. Physiotherapists found the mock consultation with the researcher (simulated patient) facilitated the transition from theory to implementation, commenting it “gave us a feel of what to expect for the upcoming pilots” and that it prevented them having to “worry about looking silly with the patients.” Similarly, the practice consultations with the pilot patients with chronic knee pain were valued, with physiotherapists reporting that this stage of practical e-learning was “hugely beneficial” and “consolidated everything” ahead of consulting with actual patients. Upon completion of the practical components of the e-learning program, physiotherapists expressed a high level of confidence and readiness to go into their first patient consultations using their new skills and knowledge. Two physiotherapists with divergent views felt that the mock consultation with the researcher was “daunting” or “confronting”, with one feeling that it was not necessary. The same physiotherapist also felt that the practice consultations with pilot patients were “surplus to needs”.

#### Theme 3: The Telehealth Journey

For the most part, physiotherapists were inexperienced in telehealth before training. Experience was largely constrained to videoconferencing for social purposes rather than for health care delivery. Physiotherapists in general were nervous or uncertain about providing care to patients via telehealth before undertaking the training program. However, 3 physiotherapists felt that they were moderately confident in telehealth before training, with one stating that “it didn’t seem like too much of a leap to deliver these services [exercise prescription via telehealth] to people.” After completing the training, most physiotherapists were much more confident and reported that they felt ready to deliver OA care via telehealth using videoconferencing. Despite improved confidence and feelings of preparedness, physiotherapists still felt that telehealth posed some challenges, particularly in supporting patients with OA to be able to navigate videoconferencing technology effectively.

#### Theme 4: The Whole Package

Physiotherapists highlighted the benefits of the structured approach of the e-learning program. They found that the combination of self-directed e-learning modules, followed by practical components (mock consultation and pilot patient consultations), was very effective as a “whole package” to develop the knowledge and skills required for best practice OA care via telehealth. Physiotherapists valued the resources and patient information booklets that accompanied the training program, stating that the resources “made it really easy” to navigate the consultations and supported the implementation of their knowledge and skills into practice. One physiotherapist with a divergent view appreciated the package but felt that they would get more out of in-person training because of their learning style.

#### Theme 5: Implementation in Broader Clinical Practice

Although the greatest knowledge gains from the training program were regarding the practical implementation of telehealth for the PEAK randomized trial, physiotherapists also described their intent to apply new knowledge and skills in OA management more broadly to their own private practice. They spoke of how the training program filled “knowledge gaps”, particularly regarding patient education, and provided them with a more structured approach to OA management that could be used when treating their own clients. Given that the training was completed before the COVID-19 pandemic, physiotherapists felt they were “well equipped for this COVID situation” and expressed feeling “ahead of the curve” when it came to implementing telehealth physiotherapy services more broadly, given social distancing and lockdown restrictions. They felt that the skills they had learned allowed them to confidently switch their private practice service delivery to telehealth as well as teach their colleagues about telehealth. Overall, 4 physiotherapists commented that despite the COVID-19 pandemic, they had not ramped up telehealth services or experienced demand for telehealth in their clinical practice.

## Discussion

### Principal Findings

**Figure 2:**
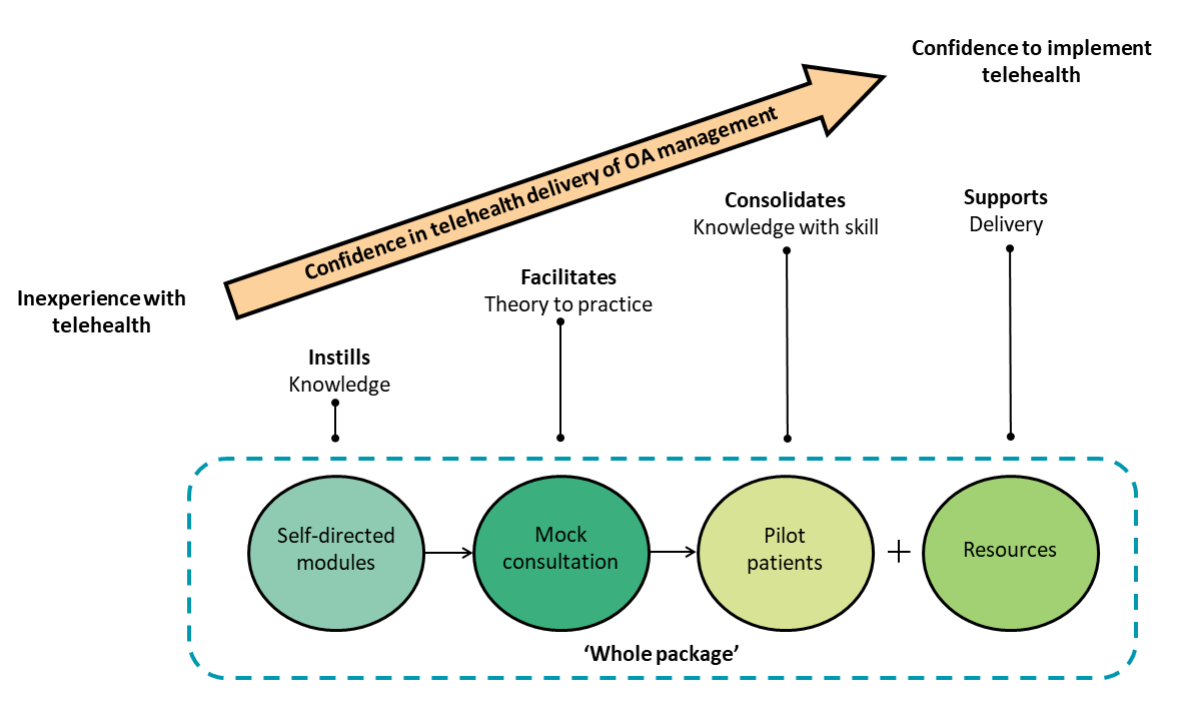
Schematic representation of emergent themes and their contribution to the development of confidence in delivering osteoarthritis care via telehealth. OA: osteoarthritis.

Our study shows that physiotherapists accepted e-learning despite their lack of familiarity with professional development delivered entirely via the internet. Physiotherapists valued both the theoretical and practical components of training, which together formed the *whole package*. Most physiotherapists described increasing confidence in providing OA management via telehealth as they progressed through the elements of the e-learning program (Figure 2). The e-learning approach first built a foundation based on new knowledge, before facilitating a transition from theory to practice, then consolidating knowledge in a clinical scenario, and ultimately resulting in the implementation of telehealth into their broader clinical practice.

Physiotherapists perceived the asynchronous aspect of the self-directed e-learning modules to be generally user-friendly; however, the unwieldy technological features of the delivery platform could be frustrating. A previous systematic review of enablers and barriers affecting e-learning in health science education found that 33% (8/24) of the studies identified the lack of user-friendly ICT as a barrier to successful e-learning [50]. This highlights the importance of a delivery platform that is simple and user-friendly when developing e-learning resources to maximize user engagement. Similarly, a previous qualitative study identified user-friendly training and technology as key factors in the successful implementation of telehealth in diabetic foot care [51]. Physiotherapists valued the comprehensive and self-paced nature of the web-based modules, which is consistent with other research. A previous study evaluated the perceptions of physiotherapy students on asynchronous e-learning for the management of chronic health conditions [52]. Students consistently highlighted the flexibility to work at their own pace and time and access to comprehensive information as advantages of e-learning. Collectively, these findings suggest that the ability to self-pace a person’s learning should be prioritized when creating adult e-learning materials.

Telehealth practical components of our e-learning program were valued by physiotherapists, which supports previous research highlighting the importance of practical components in health care education, given the need to develop *clinical or hands-on skills* alongside knowledge [53-56]. In a study evaluating e-learning for physiotherapy students on intensive care work placements [57], students perceived the e-learning modules to be helpful in preparing them for their clinical rotation and reducing their anxiety. However, e-learning modules could only sufficiently prepare students when integrated with an in-person clinical placement, where practical experience was considered necessary to build clinical reasoning skills. Similarly, other studies have noted that supervised practice and clinical simulations are valuable for building confidence and preparedness for physiotherapy students to provide care in clinical situations [58,59]. A qualitative study showed that physiotherapy students felt that clinically oriented learning, focusing on knowledge, skills, and *learning through doing*, was an important feature to include in an e-learning program about the management of chronic health conditions [52]. It is likely that the benefit of and amount of practical training required might differ depending on the level of the learner’s previous relevant clinical experience as well as the nature of the clinical skill being taught, and therefore, all of these factors should be considered when developing e-learning programs. From a pedagogical perspective, learning approaches incorporating both knowledge and skills practice are consistent with adult learning theories centered around the concept of competency being developed in sequential stages of learning, grounded in theory with proficiency built through experience [45,60,61]. Our ongoing PEAK RCT will provide additional insights into the fidelity and clinical effectiveness of the structured physiotherapy treatment plan delivered using telehealth compared with the same treatment plan delivered in person.

Although most physiotherapists described increased confidence in the delivery of telehealth after training, we do not have quantitative measures in this study documenting changes in telehealth proficiency over time. Thus, we cannot draw strong conclusions about how clinical skills in telehealth changed as a result of the training. A previous study of e-learning in physiotherapists showed that asynchronous e-learning was effective in increasing quantitatively measured confidence and knowledge in delivering self-management interventions for OA and lower back pain to patients and that the intervention was delivered with high fidelity despite a lack of supervised practical training [62]. Future research incorporating quantitative measures of intervention confidence, knowledge, and fidelity may be warranted to compare outcomes between e-learning approaches with and without synchronous practical elements of training. Future research may also aim to explore innovative alternatives to synchronous practice consultations. Such examples might include encouraging the learner to practice skills with friends or family, including completion of self-audit and self-reflection exercises.

Our sample of Australian physiotherapists was inexperienced with telehealth before training and lacked confidence in delivering care remotely. This is unsurprising given that the uptake of telehealth across Australian health services before 2020 was minimal [63,64], despite recognition of its potential to facilitate efficient health service delivery [65-67]. The COVID-19 pandemic has driven a step change in health care delivery, with rapid adoption of telehealth services by health care providers [4], coupled with increased funding for telehealth services (including physiotherapy) in several countries, including Australia [6,68] and the United Kingdom [69]. Our findings showed that the e-learning program facilitated most physiotherapists to make a rapid switch to telehealth with the sudden onset of the COVID-19 pandemic. This suggests that e-learning courses may be an effective means to train physiotherapists in best practice telehealth delivery. Education providers may also wish to incorporate e-learning telehealth training into the entry-to-practice curriculum to prepare emerging practitioners for the evolving digital health landscape.

### Strengths and Limitations

Strengths of our study include the evaluation of an e-learning program for which the e-learning modules are now freely accessible to clinicians globally. Our qualitative evaluation allowed for a thorough understanding of participants’ experiences and perceptions of participating in the e-learning program. Our interviews were conducted and the analysis was led by a person who was unknown to the participants and who was not a physiotherapist, minimizing the chances of personal or professional bias influencing findings. Limitations include that our sample was of limited size and comprised solely of physiotherapists who had applied and been selected to deliver the intervention as part of the PEAK RCT. Participants were bound by their clinical trial agreement to complete the training, and they were financially compensated for their time. Therefore, we cannot generalize our findings to the general population of physiotherapists who may be unwilling or unmotivated to complete training in their own time. As the training program is also only available in English, we cannot generalize the findings to non-English speakers nor to countries that may have a different scope of physiotherapy practice to that of Australia.

### Conclusions

In conclusion, this study provides evidence for the perceived effectiveness and acceptability of an e-learning program to train physiotherapists (in the context of a clinical trial) on best practice knee OA management, including telehealth delivery via videoconferencing. The implementation of e-learning programs to upskill physiotherapists in telehealth appears to be warranted, given the increasing adoption of telehealth service models for the delivery of clinical care.

## Appendix

Multimedia Appendix 1 Standardized competency checklist used to provide personalized verbal feedback to physiotherapists during a mock consultation with a researcher (simulated patient).

Multimedia Appendix 2 Themes, subthemes, and exemplary quotes.

## Abbreviations

ICT: information and communication technology
LMS: Learning Management System
OA: osteoarthritis
PEAK: Physiotherapy Exercise and Physical Activity for Knee Osteoarthritis
RCT: randomized controlled trial
