# Supplementary material for: Evaluation of a Novel e-Learning Program for Physiotherapists to Manage Knee Osteoarthritis via Telehealth: Qualitative Study Nested in the PEAK (Physiotherapy Exercise and Physical Activity for Knee Osteoarthritis) Randomized Controlled Trial
Source: J Med Internet Res. 2021 Apr 30;23(4):e25872. doi: 10.2196/25872 (PMC8122295; doi:10.2196/25872)
Supplement: Multimedia Appendix 1 [file jmir_v23i4e25872_app1.docx]

**Multimedia Appendix 1**

Standardized competency checklist used to provide personalized verbal feedback to physiotherapists during mock consultation with researcher (simulated patient).

| Action | Completed | Notes |
| --- | --- | --- |
| *The physiotherapist can:* | | |
| 1 Turn on the computer |  |  |
| 2 Login to the Zoom system including knowledge of password & able to communicate Meeting ID |  |  |
| 3 Dial and hang up, including ability to initiate patient in ‘waiting room’ |  |  |
| 4 Enable/Adjust settings for microphone |  |  |
| 5 Enable/Adjust settings for speaker |  |  |
| 6 Adjust camera position settings: |  |  |
| - 6a Alter angle |  |  |
| - 6b Toggle between main camera and ‘share screen’ feature |  |  |
| - 6c Instruct patient where to sit/stand in space to allow optimal assessment |  |  |
| - 6d Instruct patient how to place device/camera to allow optimal assessment |  |  |
| - 6e Use available light effectively including recommendations for patient |  |  |
| 7 Demonstrate trouble-shooting to help participant with commonly reported problems |  |  |
| 8 Access the completed pre-consultation survey (emailed PDF) to review |  |  |
| 9 Conduct a participant assessment as described in the treatment manual for an initial consultation |  |  |
| 10 Demonstrate ability to maintain RCT “blinding” integrity |  |  |
| 11 Conduct the patient education as outlined in the treatment manual for an initial consultation |  |  |
| 12 Prescribe a strengthening exercise program with appropriate intensity in accordance with study protocol, as outlined in the treatment manual for an initial consultation |  |  |
| 13 Share video clips with the patient using the “share screen’ feature of Zoom and the website of exercise videos |  |  |
| 14 Navigate the project’s password-protected exercise website |  |  |
| 15 Provide feedback to the patient about their exercise performance, including technique correction as required |  |  |
| 16 Ensure patient can use the activity tracker to record baseline daily steps |  |  |
| 17 Instruct the patient on logbook completion (for strengthening exercises, daily steps and next appointment) |  |  |
| 18 Ensures participant safety of physical environment when conducting assessment/exercise prescription |  |  |
| 19 Book the second consultation with the patient appropriately |  |  |
| 20 Record the consultation using the record feature of Zoom |  |  |
| 21 Upload the audio files to the Cloudstor Folder |  |  |
| 22 Complete the structured consultation notes and submit them to research staff |  |  |
| *Post Competency call questions* | | |
| Have you familiarized yourself with the video library and bookmarked it in your browser on the clinic computer for easy access? |  |  |
| Is your clinic receptionist aware you are participating in this trial and understands that trial booking procedures? |  |  |
| Does your clinic receptionist know to inform research staff when a patient does not attend, reschedules, or cancels an appointment (video and face-to-face participants)? |  |  |
