# Supplementary material for: Evaluation of a Novel e-Learning Program for Physiotherapists to Manage Knee Osteoarthritis via Telehealth: Qualitative Study Nested in the PEAK (Physiotherapy Exercise and Physical Activity for Knee Osteoarthritis) Randomized Controlled Trial
Source: J Med Internet Res. 2021 Apr 30;23(4):e25872. doi: 10.2196/25872 (PMC8122295; doi:10.2196/25872)
Supplement: Multimedia Appendix 2 [file jmir_v23i4e25872_app2.docx]

**Multimedia Appendix 2.**

Themes, subthemes, and exemplary quotes.

| Theme and subtheme | | Exemplary quotes |
| --- | --- | --- |
| **The experience of self-directed e-learning** | | |
|  | **High-quality, comprehensive, and user-friendly modules** | |
|  |  | Jason: “It was informative and quite concise. It wasn’t arduous. I found it quite easy to engage in, and as a participant it was a fairly natural process to go through. It wasn’t complex or confusing, it was fairly straightforward, but very informative.” |
|  |  | Brian: “Oh, it was fantastic. It was a high-quality training program which would benefit the least experienced physiotherapist and the most experienced physiotherapist. I thought it had a really good amount of content, and it definitely wasn’t overwhelming.” |
|  |  | William: “Yeah, the usability, it was user-friendly. There was no doubt about it. It was easy to navigate.” |
|  | **Unfamiliarity with professional development through e-learning** | |
|  |  | Gregory: “In terms of quality—I thought it was good. In terms of the actual type of content—obviously it was a bit different to what I’m used to. I’m used to face-to-face.” |
|  |  | William: “Well, I suppose the obvious thing with most professional development training in physio in general, it’s pretty old-fashioned...it must be face to face. So it’s really nice to have training that’s able to be delivered online and being self-paced so that you can do it if you need to.” |
|  |  | Brian: “Mostly in the past when you go and do a professional development there is a...There’s a practical component, so you’re getting in there with somebody else and doing the exercises together, or you’re practising a manual therapy technique on each other, whatever it may be. So that’s a bit more restricted when you’re doing an online learning module, or online learning modules.” |
|  | **Self-paced learning is highly valued and fits in with life** | |
|  |  | Jason: “I guess I liked being able to do it in my own time...it was quite handy as busy clinician just to be able to do it at my own pace. Compared to other PD...” |
|  |  | Anthony: “You could just jump on and do a component in the spare half an hour and then do another one later on, which was good.” |
|  |  | Leslie: “I liked that it was all self-paced, so, basically, I could do it when I had time. So I was able to get through a lot of it, I think, when I made the effort to sit down to do it, which was really good.” |
|  | **Unwieldy technological features can be frustrating** | |
|  |  | Jason: “So that was a bit annoying...it just wasn’t easy to get my fingers on that bit of information. So I found that a bit frustrating.” |
|  |  | Leslie: “I was a little bit overwhelmed to start with, having to set up logins for this and logins for that.” |
|  |  | Steven: “You couldn’t go back a couple of sections and then jump back to where you were, you had to, which just made it a little bit tedious to go through.” |
| **Practice makes perfect** | | |
|  | **The benefit of individual performance feedback** | |
|  |  | Daniel: “But it was really helpful for us to understand this is the trial run, if I make a mistake I can correct it, I’ll get a good feedback, a robust feedback which will help me.” |
|  |  | William: “I think the initial training was really good. But I’m someone who learns mostly from applying it, so the ability to have a couple of trial patients and take feedback, maybe get a feel for the delivery of the training, that way was especially really valuable.” |
|  |  | Steven: “That was actually quite useful because I recall I sort of mumbled my way through it, but he actually gave some pretty useful feedback about how to deliver some of the content.” |
|  | **Mock consult with researcher facilitated transition from theory to practice** | |
|  |  | Brian: “So that was a bit of fun. He tried to put me on the spot straight away with a troubleshooting issue, so I thought that was worthwhile. And, yeah, he kind of gave us a real feel as to what to expect for the upcoming pilots.” |
|  |  | Anthony: “It was good to do, so having someone who could kind of just briefly pause it for a second and just reinforce something that you might have not got quite right at the time. So rather than waiting till you’ve seen a few mock patients or something, like he was able to kind of correct then and then it kind of sticks in your head a bit better.” |
|  |  | Gregory: “It was good to do the practice online thing with Alex, in terms of just—more so the logistical issues, like, you know, microphone not working or the screen not working...so that was good to do it with him so you didn’t have to worry about looking silly with the patients...I think if I hadn’t had that check prior to seeing patients I would have felt a bit less prepared...So, I felt moderately prepared before the competency check and then I felt much more prepared after that.” |
|  | **Pilot patients consolidated learned skills and knowledge in a realistic scenario** | |
|  |  | Nicole: “I had a [pilot] patient that had lots of technology and language issues, so that meant that I certainly felt capable after being able to do that, once I’d had the feedback about that, then rather me thinking “What if they’re all like that.” So no, for me that was the thing that really consolidated everything else before going ahead and doing all the, you know, real patients.” |
|  |  | Edward: “even though they were pilot people—having real people actually being able to do that little bit of a better job rather than practicing fresh on them. And that was very realistic, because they were real people, very realistic...I still did forget to ask one of them something at some point and so I didn’t feel like I should just start the genuine patients straightaway. It was good to have a couple more practices where you felt like you’re not ruining the research project by messing up kind of thing.” |
|  |  | Gregory: “The pilot patients were great, in terms of practising things with them and, yeah, basically we got the knowledge and the books and all that stuff there, but then actually employing it and doing it practically can be a different thing...It was good to, not really practice on people, but to employ what we just learnt, and what we were trying to do...So I think that the pilot patients were hugely beneficial.” |
|  | **Confidence and preparedness to implement new skills** | |
|  |  | Jason. “And I think just having that practical experience flushed out any worries or nervousness about that, and improved my confidence in going ahead with the program. So I’d say after the online content I was still a bit uncertain, but then by the time I’d seen those two pilot patients I was quite happy and could see that it was going to be quite a positive experience.” |
|  |  | Caroline: “I think a combination of the session with [the researcher], and then the two pilots, yes, I think that after that, I was quite ready to see the real participants.” |
|  |  | Leslie: “Very good. Very thorough and clear and comprehensive. So I felt really well prepared going into the first consult.” |
| **The telehealth journey** | | |
|  | **Inexperienced with telehealth before training** | |
|  |  | Gregory: “Before I did the PEAK training, not very confident. I hadn’t really used video-conferencing beforehand. So, I might have used Skype or Facetime once.” |
|  |  | Jason: “I had no previous experience with the telehealth setting. And I just wasn’t sure how it’d go, but was quite impressed with it, actually.” |
|  |  | Robert: “I probably wasn’t, I don’t think I had ever really done any consultations via telehealth at that stage and so was a little bit nervous about it and nervous about doing it.” |
|  | **Significant increase in confidence and ability to deliver care remotely following training** | |
|  |  | Jason: “I would’ve been very reluctant to do it [telehealth] previously, but I think that’s...Yeah, I’m most confident to do that now...I’m 100 percent confident to attempt that with my regular private patients.” |
|  |  | Caroline: “I was definitely more confident in doing video consultations or telehealth after doing the PEAK training, so, yes, that was quite useful in my own practice.” |
|  |  | Vicki: “I’d say beforehand I could use technology, but I think the PEAK study gave a very well thought out approach to doing it via telehealth.” |
|  | **Telehealth still poses challenges after training** | |
|  |  | Steven: “Delivering via telehealth is still, I still find challenging...I guess trying to, that’s like something you can see them working but you can’t specifically ask them, you can’t look at their range of motion and that sort of thing, having them be quite distant, I suppose. So that’s challenging.” |
|  |  | Brian: “I do feel totally across it, but I guess the challenges can be just the idea of sometimes on the other end them having issues of setting up and downloading and making sure they’ve got the right software downloaded. And it can be quite stressful just navigating that, because they can be—typically when you’re dealing with an OA demographic they’re going to be older and just their ability to navigate a computer can be quite challenging. And that can be quite stressful, particularly when you’re trying to run your session within a certain time limit.” |
|  |  | Douglas: “I guess that sort of trouble shooting element with the person on the other end, when they’re struggling with technology. If I had to tick something, that’s something that can still be a challenge.” |
| **The whole package** | | |
|  | **Combination of the learning modules and applied practice was an effective learning approach** | |
|  |  | Edward: “Good going from the clunky, you know, it has to be clunky modules through to the very real pilot patients. But it did seem very practical and each step had its own use and seemed helpful to build to the point where you’re ready to deliver the research.” |
|  |  | Anthony: “So it was good to kind of go through the content, a few questions around the key concepts, do a competency check to check that, you know, you know how to use Zoom and everything else and then have a few pilot patients where you could implement the stuff you learnt straightaway in helping them. And then knowing—getting the feel for it and knowing how it all works. So, yeah, overall, I think it’s a great way to kind of do—learn and to do the whole package.” |
|  |  | Daniel: “I think it’s 10 out of 10 like, approach was 10 out of 10, that approach.” |
|  | **Patient information booklets and resources support the training package** | |
|  |  | Leslie: “I also think that all the booklets and everything are really well done, so it was very easy, clear, and concise. And I also think the way the education booklet was written is really, really good. Like I say, I think there’s a lot of tools and wording from there that I’ll continue to use in my everyday practice, because I think it was written really well.” |
|  |  | William: “I thought the material that was sent in the mail, which in particular had the cheat sheet—so you’ve a got double-sided, laminated A4 sheet of paper which talks about what to discuss in each of the sessions—that was without a doubt the most useful piece of information or content that you could’ve provided us. Which just made it really easy just to have the triggers to go through the sequencing of questioning and making sure we don’t miss out on anything.” |
|  |  | Gregory: “The books and the resistance bands and all the other stuff is well set out in that regard, it makes it a lot easier. When you get the rhythm of things and you get used to it, so right I read a book—purple book, green book, whatever it is—I thought that was, yeah, that was good in that regard too.” |
| **Implementation in broader clinical practice** | | |
|  | **Consolidating and refining existing osteoarthritis management skills** | |
|  |  | Caroline: “I think, in terms of application to my own practice, it was definitely useful to learn about what was best practice and all the different techniques that you could use or different education that you need to provide to your patients to manage the OA, so in those two settings, it was really useful.” |
|  |  | Mark: “I would say that my understanding of the education to provide has improved since undergoing the PEAK trial training. There’s some sort of knowledge gaps and little titbits that have been really helpful in doing the training.” |
|  |  | Robert: “It’s helped to mould how I now treat osteoarthritis patients by having a little bit more structure and a bit more of a process to the delivery of my treatment of those clients.” |
|  | **Enabled a switch to telehealth** **during the COVID-19 pandemic** | |
|  |  | Daniel: “One of the really proud moments I should say is when this COVID situation rolled in and we were asked to work from home. I was the only one in the unit who said that I have been delivering some programmes online on Zoom for the last two months or two and a half months or been in two pilots twice for patients. And then I’d already started with one of the other clients so I knew how we handle and how we show exercises. So I was well equipped for this COVID situation. I felt that and I was able to help others as well.” |
|  |  | Mark: “And obviously [laughs] the fact that we’re sort of in the midst of this COVID pandemic there’s much more telehealth happening outside of the trial. But I just felt like I had a head start on everyone else [laughs] in the physio world because I’ve been doing it in the PEAK trial before this hit. I felt like when the COVID pandemic really hit I was ahead of the curve.” |
|  |  | Steven: “It’s funny, actually, given obviously all the corona stuff that’s happened in the last couple of months there’s a lot of physios have sort of tried, have made that sort of shift to providing online stuff. And I guess having been through that, some sort of training prior to that, it was, it actually wasn’t too much of a...It was like, ok, well, if this is how this all has to happen then that’s ok. I can, I know, I’ve done this before, I’m sort of confident enough to still back myself to do it.” |
